# CLINICAL AND RADIOLOGICAL PARAMETERS IN MALIGNANT SPINAL TUMORS: A DESCRIPTIVE ANALYSIS

**DOI:** 10.1590/1413-785220253301e285913

**Published:** 2025-02-03

**Authors:** Marcelo Diniz de Menezes, Mariana Demétrio de Sousa Pontes, Carlos Fernando Pereira da Silva Herrero

**Affiliations:** 1 Faculdade Pernambucana de Saúde, Recife, PE, Brazil; 2 Universidade de São Paulo, Ribeirão Preto Medical School, Ribeirão Preto, SP, Brazil

**Keywords:** Neoplasms, Neoplasms, Malignant, Spine, Radiology, Clinical Epidemiology, Prevalence, Neoplasias, Neoplasias Malignas, Coluna Vertebral, Radiologia, Epidemiologia Clínica, Prevalência

## Abstract

**Objective:**

To describe the clinical and radiological parameters of spine malignant tumors.

**Methods:**

This is a therapeutic study of the descriptive retrospective type. Clinical evaluation included age, sex, tumor lesions, treatments, surgical procedures, and complications. The radiological evaluation analyzed radiographic exams, computed tomographies, and MRIs, focusing on morpho-pathological characteristics and the treatments employed.

**Results:**

Among the 236 patients evaluated, the majority were female, aged 6 to 91 years. The main complaint reported was low back pain. The most commonly used surgical approach was the posterior access, including pedicle fixation and decompression of the spinal canal. The most prevalent complication observed was infection. The majority of patients had primary breast tumors. The predominantly affected segment of the spine was the thoracic. Upon analyzing the portions of the spine affected, it was observed that the posterior arch portion was the most commonly affected.

**Conclusion:**

The clinical and radiological presentation of patients with metastatic lesions in the spine in our sample was similar to reports in the literature. Surgical outcomes aligned with previous expectations. Initial symptoms were noted, varying intervals between symptoms, diagnosis, and treatment. ***Level of Evidence III; Comparative Retrospective Study.***

## Resumo

**Objetivo:**

Descrever os parâmetros clínicos, radiológicos dos tumores malignos da coluna vertebral.

**Métodos:**

Esse é um estudo terapêutico do tipo estudo retrospectivo descritivo. A avaliação clínica incluiu idade, sexo, lesões tumorais, tratamentos e procedimentos cirúrgicos, além das complicações. A avaliação radiológica analisou exames radiográficos, tomografias computadorizadas e ressonâncias; focando em características morfopatológicas e os tratamentos empregados.

**Resultados:**

Entre os 236 pacientes avaliados, a maioria era do sexo feminino, com idades entre 6 e 91 anos. A principal queixa reportada foi a lombalgia. A abordagem cirúrgica mais utilizada foi a via de acesso posterior, incluindo fixação pelicular e descompressão do canal vertebral. A complicação mais prevalente observada foi a infecção. A maioria dos pacientes apresentava tumores primários de mama. O segmento mais frequentemente acometido da coluna vertebral foi o torácico. Ao analisar as porções da coluna vertebral afetadas, observou-se que a porção do arco posterior foi a mais comumente comprometida.

**Conclusão:**

A apresentação clínica e radiológica dos pacientes com lesões metastáticas na coluna vertebral em nossa amostra foi semelhante aos relatos da literatura. Resultados cirúrgicos alinharam-se às expectativas prévias. Nota-se a presença de sintomas iniciais, com variação nos intervalos entre sintomas, diagnóstico e tratamento. **Nível de Evidência III; Estudo Retrospectivo Comparativo.**

## INTRODUCTION

Cancer is considered the second leading cause of death worldwide, and the deficiency in early diagnosis and treatment results in 70% of these deaths occurring in low-income countries.^1^ In Brazil, it is estimated that more than 600,000 new cases of cancer are diagnosed each year. The most common type is skin cancer, followed by breast cancer in women and prostate cancer in men.^2^ Stemming from primary cancerous lesions, metastatic lesions have been showing an increasing incidence, with the spinal column being the most commonly affected site by skeletal metastasis.^3^ Previous studies have identified that approximately 70% of cancer patients present some metastasis in the spinal column and that 40% of deceased patients did not exhibit symptoms resulting from the lesion.^4^

The diagnosis of the primary lesion can vary greatly, with the most common being prostate, breast, melanoma, lung, and kidney cancers^3^.

However, other carcinomas are also less commonly associated with spinal column metastases, such as gastrointestinal tumors and thyroid carcinoma.^3^ Additionally, metastatic tumors of the spinal column have a slightly higher prevalence in men compared to women, due to the high incidence of bone involvement in prostate cancer.^3^,^4^,^5^ All age groups can be affected, with a higher prevalence among individuals aged 40 to 65 years.^3^

The optimal treatment for patients with metastatic spinal cord injury involves a multidisciplinary approach, and while the therapeutic advances currently used yield better results, many remain as palliative goals, reducing morbidity and improving patients’ quality of life.^6^ Thus, although often not used as curative, spinal surgery can play an important role in treating patients with mechanical instability, progressive tumor growth, pain unresponsive to clinical measures, and neurological symptoms.^7^

The main objective of the present study is to conduct a descriptive epidemiological study of a sample of patients with spinal metastasis who underwent surgical treatment at a tertiary hospital in the public healthcare system of a low-income country. Additionally, the secondary objective emphasizes the initial symptoms of patients, as well as the time until the diagnosis of the metastatic lesion and subsequent surgical treatment.

## METHODS

### Study Protocol

This is a single-center study that involved the descriptive use of a prospective database. A total of 236 patients with confirmed spinal metastasis were included in the study and underwent surgical treatment by a group of spine surgeons at a tertiary-level public healthcare hospital in Brazil, between the years 2005 and 2017.

Ethical Considerations

The research project was approved by the Research Ethics Committee of the Institution where the study was conducted, including the waiver of the Informed Consent Form, since the use of patient data was completely anonymized and the data collection was not influenced by any treatment decisions established (protocol HC 354/2018—CAAE: 82389518.0.0000.5440).

### Patient sample

The inclusion criteria used comprised patients with a confirmed diagnosis of metastatic lesions in the spinal column, histopathologically confirmed, undergoing treatment for vertebral metastases, of both sexes, of any race and age group. The exclusion criteria included the absence of a confirmatory diagnosis of metastatic disease in the spinal column and patients who did not undergo surgical treatment.

### Data collection (studied variables)

The data were obtained by the researchers from the Medical Records Department (MRD) of the hospital, and both medical annotations and complementary exams were utilized. The clinical variables used included patient gender; age at diagnosis; patient symptoms and the time between symptom onset and definitive diagnosis of metastatic disease; the time between diagnosis and surgical treatment of the spinal column; neurological manifestations; surgical access route and techniques employed; postoperative complications; and the need for further surgical intervention. Evaluation of complementary exams involved the use of data from histopathological exams and assessment of radiological exams such as plain radiography and magnetic resonance imaging. Variables obtained from imaging exams included morpho pathological characteristics of the lesion: affected vertebral segment and level, and the portion of the vertebral body involved in the metastatic lesion.

### Statistical Analysis

The parameters were stored in a Microsoft Excel spreadsheet, and the results were presented as percentages, means, and medians.

## RESULTS

Out of the total of 236 patients included in the study from 2005 to 2017, 105 (44.49%) were male, and 131 (55.5%) were female. The age of the patients at the time of diagnosis ranged from 6 to 91 years, with a mean of 55 years. The spinal-related symptoms presented included neck pain in 14 (5.96%) patients, low back pain in 60 (25.42%), and thoracic pain in 30 (12.71%) patients. Regarding neurological manifestations, 122 (51.7%) patients had a partial deficit, and 22 (9.32%) had a complete deficit at the time of the initial evaluation.

The study of the time between the onset of spinal-related symptoms and the diagnosis of metastasis by magnetic resonance imaging revealed results ranging from 1 day to 9 years, with a mean of 70.5 days and a median of 73.5 days. The time between diagnosis and surgical treatment of the spinal column varied from 0 days to 5 months and 15 days.

Regarding the surgical procedures performed, 210 (88.98%) patients underwent a posterior approach, 14 (5.93%) patients underwent an anterior approach, and 12 (5.08%) underwent a combined approach (anterior and posterior). Of the patients undergoing a posterior approach, all were treated with pedicle fixation and vertebral canal decompression, and among these, 51 (21.62%) patients additionally underwent corpectomy and replacement with an interbody device filled with bone cement. Additionally, kyphoplasty was the technique of choice used in 5 (2.38%) patients operated on via the posterior approach. No patient underwent an isolated anterior approach, nor a combined approach with pedicle fixation and posterior decompression associated with corpectomy and replacement with an interbody device filled with bone cement. Seventy-one (30%) patients experienced postoperative complications, with 31 (43.66%) patients diagnosed with infection, 3 (4.22%) patients with extradural hematoma, 3 (4.22%) patients with seroma or wound dehiscence, and 3 (4.22%) patients with worsened neurological deficit. Thus, 60 (25.42%) patients required a new surgical approach to treat such complications related to the initial procedure, and 2 (0.84%) patients underwent a new procedure due to tumor recurrence and resulting new symptomatic compression.

The findings of the histopathological examination revealed 39 (16.52%) patients diagnosed with metastasis resulting from primary breast cancer, such as mammary sarcoma, invasive ductal carcinoma of the breast, breast adenocarcinoma, high-grade pleomorphic mammary sarcoma, and inflammatory breast carcinoma.

The spinal column segment most affected by metastatic lesions was the thoracic segment in 123 (52.11%) patients, followed by the lumbar segment in 83 (36.16%) patients, cervical segment in 40 (16.94%) patients, and sacral segment in 13 (5.50%) patients. The most affected vertebral portion by the tumor lesion was the posterior arch portion in 183 (77.54%) patients, followed by the vertebral body in 183 (77.54%) patients.

## DISCUSSION

In Brazil, most hospitals treating patients diagnosed with cancer are not specialized and exclusive centers, especially when it comes to public healthcare facilities.^8^ Thus, although accredited for high-complexity treatments, the hospital where our study was conducted is not an exclusive referral center for the treatment of cancer patients. This may explain why the epidemiological findings differ from studies previously published by specialized cancer treatment centers and their associated complications.^8^

About the methodology employed in the study, although retrospective, the patients’ data were adequately obtained from detailed descriptions in the medical records of the included patients, which were available in the Medical Records Department (MRD) of the hospital. Thus, the results regarding the number of female patients (131) being greater than the number of male patients (105) are consistent with data previously published in the literature. Regarding the age of the patients at the time of diagnosis, which ranged from 6 to 91 years, our findings are in line with the literature, which indicates that the incidence of spinal metastatic lesions increases with age, being more common in patients around the fifth decade of life.^8^,^9^

As expected, the most prevalent primary cancer diagnosis was breast cancer in 37 (15.67%) patients, followed by prostate cancer in 27 (11.44%) patients, which also followed results previously published by other studies.^10^,^11^,^12^ The thoracic spine was the segment most affected by secondary lesions, with 123 (52.11%) patients, followed by the lumbar segment, with 83 (36.16%) patients, and cervical, with 40 (16.94%) patients. This finding corroborates the results of the literature, where thoracic spine metastases account for 70%, and lumbar spine for 20%.^8^ Similarly, the most affected vertebral portion in our patient sample was the posterior portion in approximately 77% of patients, followed by the vertebral body in nearly 34% of patients, values similar to those established by other studies.^11^

The occurrence of pain symptoms resulting from neglected metastatic lesions by healthcare professionals is a relevant aspect that corroborates our findings.^13^ In our study, pain was present in 218 (92.37%) patients, while neurological deficit was present in 144 (61%) patients. Despite this, the time between the initial symptom and the diagnosis of the metastatic lesion ranged from 1 day to 9 years, which may impact patient treatment.^8^

Regarding the outcomes of surgical treatment, previous literature data indicate neurological recovery in 22.7% of patients and worsening in 2.2% of patients.^8^ In our study, 66 (28%) patients showed improvement in neurological function, while 19 (8.05%) patients experienced worsening of the deficit. The study did not aim to evaluate the use of neoadjuvant or adjuvant therapy, which should be considered in each particular case by the multidisciplinary team. The choice of surgical treatment for the metastatic lesion took into account factors already described in the literature such as the primary tumor, location of the lesion in the spinal column, and the patient’s overall condition.^14^ Thus, in many cases, there may be a period between the diagnosis of the lesion and the performance of surgical treatment,^8^ which can explain why our results regarding the time between the diagnosis of the lesion and the establishment of surgical treatment have varied from 0 days to 5 months and 15 days.

The surgical technique considered ideal is one that adequately exposes the lesion and safely removes it.^15^ The choice of surgical technique was at the discretion of the hospital’s spine surgery team where the study was conducted and relied on the principles of spinal canal decompression (81.35%), followed by surgical stabilization with pedicle fixation (81%), and when necessary, corpectomy associated with vertebral body replacement by an interbody device filled with bone cement (21.62%). Furthermore, 19 (8%) patients underwent vertebral body kyphoplasty procedure, which did not present complications, as it is a rapidly performed procedure and does not require long post-surgical hospitalization periods.^15^

Regarding complications arising from surgical treatment, a literature review^16^ showed that the complication rate of surgical treatment for spinal metastases ranged from 10% to 52%. In the present study, 71 (30%) patients experienced postoperative complications, and 60 (25.42%) patients required further surgical intervention. These findings are consistent with the understanding that surgeries involving pedicle fixations associated with vertebral body resection and subsequent replacement have a higher rate of complications.^11^ An important detail worth mentioning is that during the study period when the patients were treated, the percutaneous pedicle fixation technique had not been implemented in the hospital where the study was conducted. We are aware that currently percutaneous fixation is an additional available tool in the treatment of patients with metastatic spinal column lesions and may yield different results from those found in our study, as it is a less invasive technique.^8^

This study has limitations that deserve mention. Despite the quality of the medical documentation from which the data were obtained, it is a retrospective study at a single high-complexity treatment center. Thus, the data regarding the prevalence of each primary lesion may be subject to sampling bias. Nevertheless, our findings were consistent with data previously published in the literature. Another limitation is the lack of specific information about the reasons why each patient waited between the diagnosis of spinal metastatic lesions and surgical treatment, which would provide crucial insights to enhance the treatment of these patients. On the other hand, we have demonstrated the importance of early symptoms and the need for greater attention to optimize diagnosis and initiate treatment promptly, despite the development of complementary technologies.

## CONCLUSION

In our sample, the clinical and radiological presentation of patients with spinal metastatic lesions varied but was similar to those previously reported in the literature, as were our surgical treatment outcomes for these patients. However, it is important to highlight the presence of early symptoms and the variable time for diagnosis and treatment of the patients.

## References

[1] American Cancer Society (2019). Cancer facts & figures 2019 [Internet].

[2] Instituto Nacional de Câncer (Brasil) (2019). Institucional.

[3] Araujo JL, Veiga JC, Figueiredo EG, Barboza VR, Daniel JW, Panagopoulos AT (2013). Management of metastatic spinal column neoplasms--an update. Rev Col Bras Cir.

[4] Wong DA, Fornasier VL, MacNab I (1990). Spinal metástases; the obvious, the occult, and the impostors. Spine.

[5] Shobeiri P, Seyedmirzaei H, Kalantari A, Mohammadi E, Rezaei N, Hanaei S (2023). The Epidemiology of Brain and Spinal Cord Tumors. Adv Exp Med Biol.

[6] Gilbert RW, Kim JH, Posner JB (1978). Epidural spinal cord compression from metastatic tumor: diagnosis and treatment. Ann Neurol.

[7] Quraishi NA, Gokaslan ZL, Boriani S (2010). The surgical management of metastatic epidural compression of the spinal cord. J Bone Joint Surg Br.

[8] Alexandre M, Santos WZ, Góes R, Gotfryd AO, Topolniak R, Meves R (2022). Profile of patients with spine tumor operated in a South America Reference Service: epidemiological study. Coluna/Columna.

[9] Chi JH, Bydon A, Hsieh P, Witham T, Wolinsky JP, Gokaslan ZL (2008). Epidemiology and Demographics for Primary Vertebral Tumors. Neurosurg Clin N Am.

[10] Daniel JW, Veiga JCE (2007). Diretrizes no tratamento das metástases epidurais da coluna vertebral. Atualização. Arq Bras de Neurocir.

[11] Pontes MD, Pires BPF, Albuquerque FP, Herrero CFS (2020). Assessment of the posterior approach for surgical treatment of spinal metastatic breast cancer. Acta Ortop Bras.

[12] Instituto Nacional de Câncer (Brasil) (2019). Estimativa 2020: Incidência de Câncer no Brasil.

[13] Perrin RG, Laxton AW (2004). Metastatic spine disease: epidemiology, pathophysiology, and evaluation of patients. Neurosurg Clin N Am.

[14] Tokuhashi Y, Matsuzaki H, Oda H, Oshima M, Ryu J (2005). A Revised Scoring System for Preoperative Evaluation of Metastatic Spine Tumor Prognosis. Spine (Phila Pa 1976).

[15] Molina C, Goodwin CR, Abu-Bonsrah N, Elder BD, De la Garza Ramos R, Sciubba DM (2016). Posterior approaches for symptomatic metastatic spinal cord compression. Neurosurg Focus.

[16] Shehadi JA, Sciubba DM, Suk I, Suki D, Maldaun MV, McCutcheon IE (2007). Surgical treatment strategies and outcome in patients with breast cancer metastatic to the spine: a review of 87 patients. Eur Spine J.

